# Intraoperative fluoroscopy skills in distal radius fracture surgery: valid and reliable assessment on a novel immersive virtual reality simulator

**DOI:** 10.2340/17453674.2024.41345

**Published:** 2024-08-28

**Authors:** Marie SØNDERUP, Amandus GUSTAFSSON, Lars KONGE, Mads Emil JACOBSEN

**Affiliations:** 1 Department of Clinical Medicine, Faculty of Health Science, University of Copenhagen; 2 Copenhagen Academy for Medical Education and Simulation (CAMES), Rigshospitalet; 3 Department of Orthopedic Surgery, Center for Orthopedic Research an Innovation (CORI), Næstved Slagelse Ringsted Hospitals, Denmark

## Abstract

**Background and purpose:**

Orthopedic trainees must be able to perform intraoperative fluoroscopy imaging to assess the surgical result after volar locking plate surgeries of distal radius fractures. Guided by Messick’s contemporary validity framework, the aim of our study was to gather evidence of validity for a test of proficiency for intraoperative imaging of a distal radius fracture using a novel immersive virtual reality simulator.

**Methods:**

11 novices and 9 experienced surgeons employed at orthopedic departments completed 2 individual simulator sessions. At each session the participants performed 3 repetitions of an intraoperative fluoroscopic control of a distal radius fracture, consisting of 5 different fluoroscopic views. Several performance metrics were automatically recorded by the simulator and compared between the 2 groups.

**Results:**

Simulator metrics for 3 of the 5 fluoroscopic views could discriminate between novices and experienced surgeons. An estimated composite score based on these 3 views showed good test–retest reliability, ICC = 0.82 (confidence interval 0.65–0.92; P < 0.001). A discriminatory standard was set at a composite score of 6.15 points resulting in 1 false positive (i.e., novice scoring better than the standard), and 1 false negative (i.e., experienced surgeon scoring worse than the standard).

**Conclusion:**

This study provided validity evidence from all 5 sources of Messick’s contemporary validity framework (content, response process, internal structure, relationship with other variables, and consequences) for a simulation-based test of proficiency in intraoperative fluoroscopic control of a distal radius fracture fixated by a volar locking plate.

Distal radius fractures (DRFs) are among the most common fractures. For those DRFs requiring surgery, plate fixation is reported to account for 48–96% of surgeries performed [1,2]. When performing open reduction and internal fixation (ORIF) of DRFs, the use of intraoperative fluoroscopy is essential to ensure accurate fracture reduction and appropriate implant positioning to reduce the risk of complications [3-5]. Also, a significant inverse association between the quality of intraoperative fluoroscopic control images and patients’ subsequent risk of reoperation has been shown [6].

Despite its importance, there is a noticeable lack of formal training of orthopedic trainees in intraoperative fluoroscopic skills [3,7]. Virtual reality simulation training has been proposed to mitigate the impact of surgeons’ early learning curve on patient outcomes, and indeed technology-enhanced simulation-based training has proven highly effective in skills acquisition [8]. Computer-assisted simulators that employ fluoroscopy exist, e.g., in hip fracture surgery, but these simulators use standardized images in ideal projections for training purposes [9,10]. Consequently, they do not offer training in the acquisition of ideal images, which is a critical aspect of fluoroscopy. To our knowledge, no commercially available simulators exist for the systematic training of intraoperative fluoroscopy imaging skills, either in a general context, or specifically for volar locking plate fixation of DRFs.

To address this educational gap, we have developed an immersive virtual reality (iVR) simulator in collaboration with software developer VitaSim (www.vitasim.dk). The iVR simulator enables practice without exposure to radiation or compromising patient safety and could help ensure basic competency of trainees before they proceed to perform intraoperative imaging in the operating room.

Prior to the implementation of simulator training and testing, it is imperative to establish supporting validity evidence for the simulator test, established through a rigorous validation process informed by a contemporary validity framework [11]. The aim of this study was to gather validity evidence from all 5 sources in Messick’s validity framework: content, response process, internal structure, relationship with other variables, and consequences [12].

## Methods

The study was conducted from March 2023 to September 2023 at 3 different sites: (i) Copenhagen Academy for Medical Education and Simulation (CAMES), Denmark, (ii) Hand Clinic, Orthopaedic Department, Herlev and Gentofte University Hospitals, Denmark, and (iii) Orthopaedic Department, Slagelse Hospital, Denmark.

### Participants

Participants were novices or experienced surgeons recruited through invitation. Novices were medical doctors who responded to an open invitation sent through a social media group for physicians at orthopedic departments in Eastern Denmark. All novices were employed at orthopedic departments, either as interns or in their 1st year of orthopedic specialization, corresponding to PGY 1–2. The exclusion criterion for novices was prior performance of more than 15 volar locking plate surgeries under supervision. This criterion was chosen to encompass the variability in experience, from complete novices to beginners, among junior trainees. Experienced surgeons were consultants in either orthopedic trauma surgery or hand surgery with prior performance of at least 100 volar locking plate surgeries. All participants filled out a demographics questionnaire and, in addition, the experienced surgeons completed a questionnaire regarding which intraoperative fluoroscopy images they routinely perform for documentation after volar plate fixation of DRFs. All participants were tested on the same simulator, in the same settings, and all participation was voluntary.

### iVR simulator

**Figure 1:**
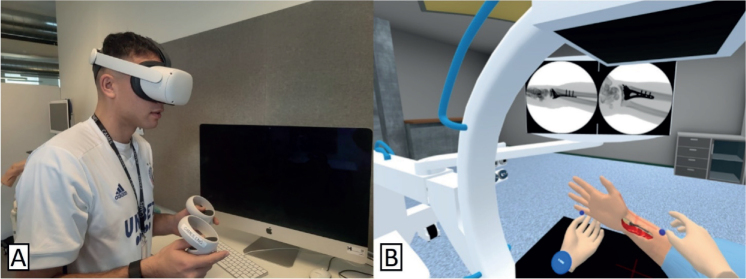
(A) Illustration of a surgeon using the simulator consisting of a head-mounted display and 2 handheld controllers, and (B) illustration of the simulated scenario from the users’ perspective.

The iVR simulator used in this study consists of a head-mounted display (Oculus Quest 2 [Meta, Menlo Park, CA, USA]) connected to a laptop (Lenovo Legion 5i; www.lenovo.com/en), and 2 handheld controllers (Oculus Touch) (Figure 1). In the simulated scenario, the user is immersed in an operating room with a C-arm fluoroscopy unit and a patient who has undergone volar plating surgery. In the simulator software a 3D model of the bony parts of an upper extremity was created based on rendering of a computed tomography scan. By use of ray-tracing technology in the virtual reality software Unity (www.unity.com) realistic fluoroscopy images are created depending on the position of the wrist relative to the fluoroscopy machine. In this scenario, the patient is lying in the supine position on an operating table with the right upper extremity on an arm table (Figure 1). The user can move the patient’s right arm and hand freely along 3 different axes: (i) elbow flexion and extension, (ii) forearm rotation, and (iii) wrist flexion and extension. The user can make and save an unlimited number of fluoroscopy images. Images that are not saved are deleted when a new image is made. When concluding the scenario, the user is prompted to transfer a total of 5 images for documentation, from the pool of saved images, corresponding to 5 pre-defined fluoroscopy views, as defined in a recently published global Delphi consensus study [13]. These 5 fluoroscopy views were: (i) posteroanterior (PA) view (palm towards the X-ray detector), (ii) anteroposterior (AP) view (palm towards the X-ray source), (iii) straight lateral view, (iv) lateral facet view (elevated wrist), and (v) a dorsal tangential view. A video demonstrating an iVR simulator session can be found with the online version of this manuscript. For each of the images transferred for documentation, angular values for elbow flexion and forearm rotation, in relation to the C-arm, were automatically recorded by the simulator. For the tangential image, the angle of the wrist relative to the patient’s forearm was additionally recorded. The total number of images taken as well as the time taken to complete the task were automatically recorded for all repetitions.

### Study design

Participants took part in 2 individual iVR simulator sessions with a minimum of 4 weeks between sessions. During the 1st session, all participants received a standardized introduction to the study and equipment followed by a standardized “warm-up” exercise to familiarize themselves with all functionalities of the simulator. Subsequently, the participants were instructed to perform 3 repetitions of a complete intraoperative fluoroscopy control of a DRF fixated by a volar locking plate consisting of all 5 images. The 2nd session was identical to the 1st, except there was no introduction and warm-up exercise. During all simulations, predefined help with simulator functionalities was provided upon request from the participant, but no other help or feedback was provided.

### Statistics

All angular scores were converted into absolute z-scores, which were normalized based on the mean scores of the group of experienced surgeons across all repetitions. We then computed a total z-score for each image by summing the associated angular z-scores. Subsequently, we identified simulator metrics (i.e., time to complete, total number of images taken, and total z-scores) with discriminatory ability between experience levels by employing separate 2-way mixed analyses of variance (ANOVA) with the simulator metric as the repeated-measures variable and experience as the between-group variable. To control for familywise error rate in multiple testing, we applied the Holm–Bonferroni correction. Finally, we combined the total z-scores for outcomes that could discriminate between groups into a single composite score for each repetition of each participant.

We explored the internal structure of the simulator test in 2 ways: (i) by visually comparing estimated marginal means for the composite scores across the 2 groups for the 2 sessions, and (ii) by analyzing test–retest reliability for all composite scores calculating a 2-way mixed intraclass correlation coefficient (ICC) with the absolute agreement definition. We analyzed relations to other variables (i.e., the ability of the composite score to discriminate between the 2 groups) by 2-way mixed ANOVA with composite score as the repeated-measures variable and experience as the between-group variable. Subsequently, we calculated a mean composite z-score over the 6 repetitions for each participant. We then plotted the distribution of these mean composite z-scores for the 2 groups using the contrasting groups’ standard setting method [14]. The intersection between the groups was set as a discriminatory standard, and the consequences of this standard were explored.

We employed a listwise exclusion of participants to handle missing data, consistent with the assumptions of the statistical models employed. The composite z-scores, i.e., performance scores, that we report are estimated marginal (EM) means. We considered differences between groups as statistically significant when the P was < 0.05, unless otherwise stated. We used 95% confidence intervals (CI). All data was analyzed using IBM SPSS statistics software (version 29, IBM Corp, Armonk, NY, USA).

### Ethics, funding, and disclosures

This study was deemed exempt from ethical approval by the Regional Ethical Committee of the Capital Region of Denmark (F-22074325, dated 09-01-2023). Prior to enrollment, all participants provided written informed consent. This study was supported by a research grant for the 1st author by the Novo Nordisk Foundation. Development of the iVR simulator was supported by grants from Toyota-Fonden, Denmark (grant number KJ/BG-10053H), Frimodt-Heineke Fonden, and Tømmerhandler Johannes Fogs Fond (grant number 2023-0252). Further, the software developer financed 50% of simulator development costs. Funders have not had any influence on any aspect of study design, data collection, data analysis, interpretation of results, or manuscript preparation. Intellectual property rights for the iVR simulator software are shared equally by the software developer and the Capital Region of Denmark. The authors of this study have no financial interests to declare. Complete disclosure of interest forms according to ICMJE are available on the article page, doi: 10.2340/17453674.2024.41345

## Results

**Figure 2:**
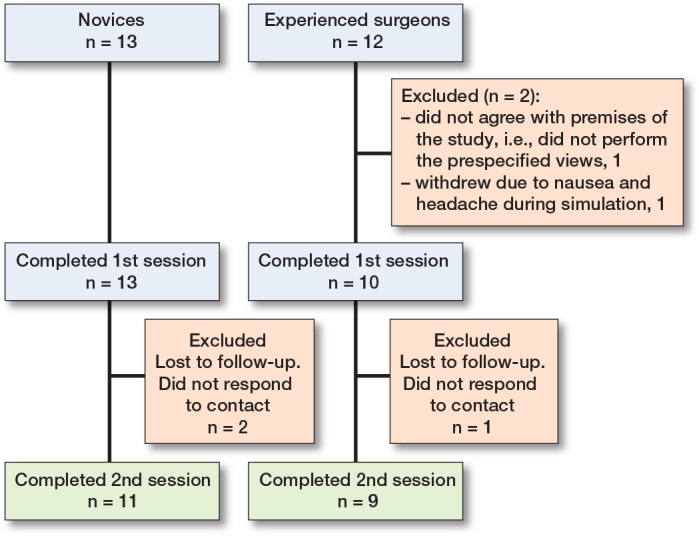
Flowchart of participants.

25 participants were enrolled in the study. 13 were novices, representing 8 different orthopedic departments, of whom 11 completed both sessions. 12 were experienced surgeons, from 2 different orthopedic departments, of whom 9 completed both sessions (Figure 2). Participants were similar as to sex and handedness. Experienced surgeons were older and tended to have more days between their simulator sessions (Table 1). All sessions by all participants were concluded in less than 25 minutes. All the experienced surgeons routinely performed PA view and lateral facet view for fluoroscopy control, 4/9 routinely performed a straight lateral view, whereas only 1/9 routinely performed PA view and tangential, respectively.

**Table 1:** Participant characteristics

Factor	Novices n = 11	Experienced surgeons n = 9
Age, median (IQR)	30 (28–34)	48 (40–51)
Sex, female:male, n	5:6	3:6
Dominant hand, right:left:ambidextrous, n	10:0:1	9:0:0
Days between sessions, median (IQR)	38 (32–47)	41 (33–102)
Uses glasses, yes, n	2	6
Months as orthopedic intern/resident, median (IQR)	9 (8–15)	–
Years as an orthopedic surgeon, median (IQR)	–	5.5 (2.5–11.5)
Supervised volar plating procedures, n, median (IQR)		
overall	4 (0–6)	–
between sessions 1 and 2	1 (0–2)	–
Experienced surgeons’ subspeciality		
orthopedic trauma surgery:hand surgery, n	–	1:8

**Figure 3:**
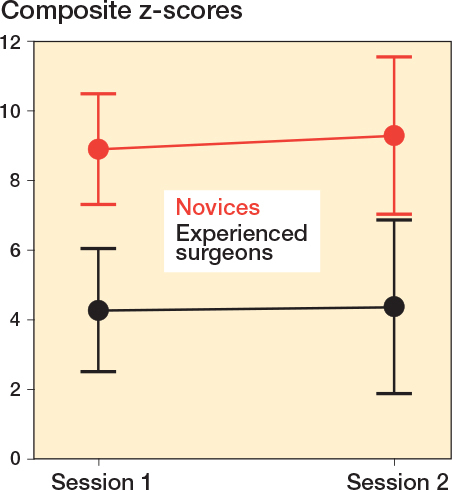
Composite z-scores, expressed as estimated marginal means, across the 2 sessions for the novices (red) and experienced surgeons (black). Error bars represent the estimated 95% confidence intervals.

**Figure 4:**
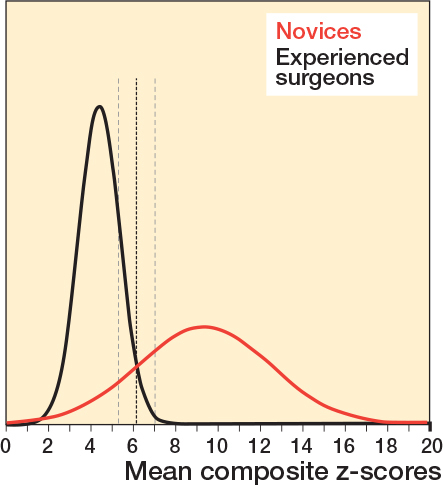
Distribution of mean composite z-scores across all 6 repetitions for novices (red) and experienced surgeons (black). The bold dotted line represents the intersection of the curves determining the discriminatory standard using the contrasting groups’ method and the thin dotted lines represent the lower and upper borders of the 95% CI of the intersection.

**Figure 5:**
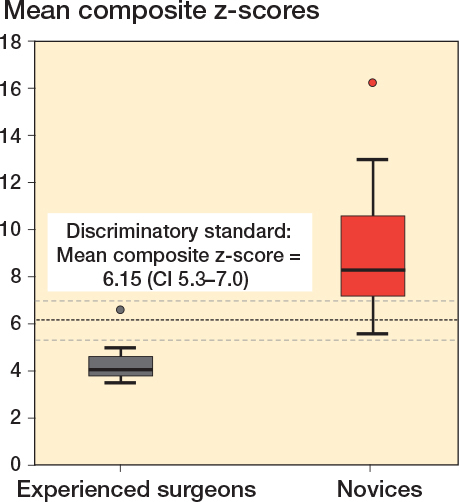
Box plot illustrating mean composite z-scores across all 6 repetitions for novices (red) and experienced surgeons (grey). The bold dotted line represents the discriminatory standard. 1 novice performed better than the defined standard and 1 experienced surgeon, who was an outlier, performed worse than the defined standard.

There was a statistically significant difference between the 2 groups for total PA image z-score, total lateral image z-score, and total facet image z-score, respectively, indicating discriminatory ability of the simulator test for these projections (Table 2). These 3 metrics were combined to a single composite z-score. There was no difference in estimated mean composite z-scores over the 2 sessions as the estimated mean composite z-score difference for all participants was 0.43 (CI –0.98 to 1.85), indicating no difference in performance for either of the 2 groups across the 2 sessions (Figure 3). The estimated composite z-score showed good test–retest reliability with ICC for average measures being 0.82 (CI 0.65–0.92), P < 0.001. Estimated mean composite z-scores were 4.39 (CI 2.7–6.08) for the experienced surgeons and 9.34 (CI 7.81–10.87) for the novices, P < 0.001, with a very large effect size, partial eta squared = 0.54 indicating a large difference in performance scores between groups. Using the contrasting groups’ method, we defined a discriminatory standard for the mean composite score as 6.15 (CI 5.3–7.0) (Figure 4). The consequences of this discriminatory standard were that all but 1 of the novices scored worse than the standard and all but 1 of the experienced surgeons scored better than the standard (Figure 5).

**Table 2:** Performance scores for the 2 groups for each simulator metric

Simulator metrics	Novices n = 11 EM mean ^a^ (CI)	Experienced surgeons n = 9 EM mean ^a^ (CI)	Adjusted significance level ^b^	P	Effect size ^c^
Total z-score					
lateral view	2.52 (2.14–2.91)	1.56 (1.13–1.99)	0.007	0.002	0.41
facet view	4.48 (3.16–5.79)	1.51 (0.06–2.96)	0.008	0.005	0.36
PA view	2.34 1.(85–2.83)	1.33 (0.79–1.87)	0.01	0.009	0.32
tangential view	3.07 (2.40–3.76)	2.13 (1.38-2.89)	0.01	0.07	0.18
AP view	1.54 (1.20–1.88)	1.65 (1.28–2.03)	–	0.7	0.011
Time, s	210 (165–255)	229 (179–279)	–	0.6	0.02
No. of images	24.5 (16.4–32.5)	23.9 (15.0–32.7)	–	0.9	0.001

a Estimated marginal mean.

b Adjusted significance levels using the Holm–Bonferroni correction to adjust for familywise error rate in multiple testing.

c η²p (partial eta squared).

## Discussion

**Figure 6:**
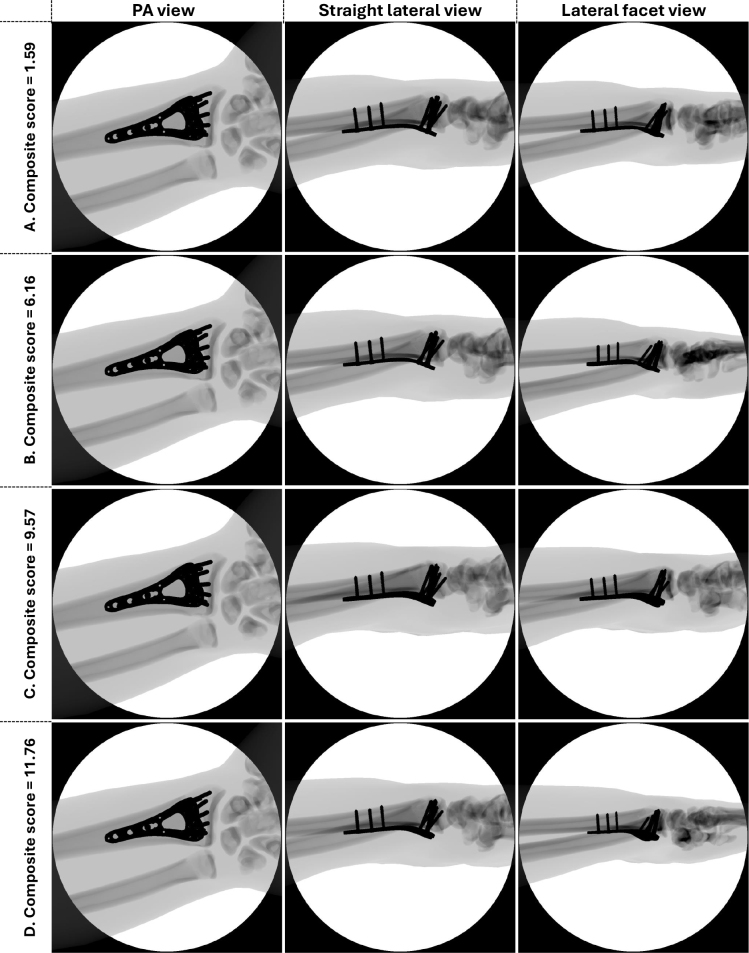
Examples of image series and corresponding composite scores from the simulator. A. Note DRUJ in the PA view. In both lateral views the plate is in profile. The radiocarpal joint is correctly visualized in the facet view. B. In the facet view the joint is double contoured. C. DRUJ not visualized in the PA view. The plate is not in profile in either of the lateral views, and the facet view cannot with certainty rule out that the distal most radial screw is intraarticular. D. Plate not in profile in the lateral view and the radiocarpal joint line is not adequately visualized in the facet view. Series A scored better than the discriminatory standard, and B through D scored worse than the discriminatory standard.

The study aimed to gather validity evidence from all 5 sources (content, response process, internal structure, relationship to other variables, and consequences) of Messick’s contemporary validity framework for an iVR simulator test to assess proficiency in intraoperative imaging of a DRF fixated with a volar locking plate. We found validity evidence from all 5 sources to support the interpretation of test scores (Table 3, see Appendix). Further, a credible discriminatory standard for the test was established [15]. Higher test scores (i.e., worse performance) resulted in lower image quality, supporting the clinical relevance of the simulator test (Figure 6, see Appendix).

**Table 3:** Validity evidence according to Messick’s validity framework

Source of evidence	Question related to each source of evidence	Validity evidence
Content	Does the content of the test reflect the construct it is intended to measure?	Tasks are aligned with the construct: The images to assess were defined by an international expert consensus [13].
Response process	What have been done to reduce bias?	The participants received standardized instructions and were tested in the same setting. The simulator provided objective metric scores.
Internal structure	Is the test score reliable?	High level of reliability: No difference in mean composite scores for both groups across sessions (Figure 3). Good test-retest reliability measured by ICC.
Relationship with other variables	Is there a correlation between performance score and a recognized measure of competency?	Experienced surgeons statistically significantly outperformed the novices in the 3 fluoroscopy projections they reported performing routinely (Table 2).
Consequences	What are the consequences of the d iscriminatory standard?	1 of 11 novices performed better than the defined standard and 1 of 9 experienced surgeons performed worse than the standard (Figures 4 and 5).

In our study, 3 out of 5 fluoroscopic views showed statistically significant differences in total z-scores between experienced surgeons and novices. A composite score from these 3 views was highly discriminatory between the 2 groups. Unexpectedly, 2 views failed to differentiate between experience levels, despite being identified as essential in a global Delphi consensus study by 87 orthopedic experts for assessing DRF fixation with a volar locking plate [13]. However, these 2 non-discriminatory views were not routinely used by the experienced surgeons in our sample, indicating that their expertise did not extend to these specific projections. Consequently, this finding reinforces the validity argument for the simulator test as a measure of domain-specific proficiency rather than just general surgical experience. Nevertheless, studies have found that several fluoroscopic views are necessary to assess the surgical result after volar plating of a DRF. This includes a tangential view, shown to be superior to the lateral view in detecting dorsal screw penetration [5,16,17]. Further, the AP view is commonly the standard projection for postoperative radiographic control images and is therefore valuable for serial assessment of intra- and postoperative images [18]. Accordingly, although the simulator test for these 2 views could not discriminate between groups in our sample of participants, we believe that an ensuing simulation-based curriculum should include them.

The literature on intraoperative fluoroscopy predominantly addresses 2 themes: characterization and quantification of radiation exposure for specific surgeries and operators of different surgical experience, and exploration of interventions to reduce radiation exposure. Annual doses of radiation to which orthopedic surgeons are subjected are generally below recommended threshold levels [19]. The cumulative radiation exposure endured through an entire career in orthopedics could, however, increase the risk of cancer and other associated diseases [20]. Moreover, surgeons of lesser experience are reported to utilize larger radiation doses by fluoroscopy than their more experienced colleagues [21]. As such, studies investigating methods to reduce radiation exposure are both important and in demand as occupational risks should be minimized wherever feasible. Unsurprisingly, educational interventions aimed at enhancing radiation exposure awareness indeed led to reduced exposure [22–24]. We argue that while focusing on radiation safety through education is vital, equal emphasis must be placed on mastering fluoroscopic techniques to maintain high-quality care and ensure patient safety. In fact, enhancing skills in fluoroscopy could lead to reduced radiation exposure, as a deeper understanding of the information each fluoroscopic view provides is essential for minimizing exposure without overlooking critical issues. In line with these considerations, Rikli et al. [7] introduced a performance improvement program, consisting of a 21-minute video and a poster, to improve resident surgeons’ abilities to obtain correct lateral views in hip fracture surgery. By means of a pragmatic evaluation method, they found not only post-intervention improvement in lateral view quality, but also improved reduction and implant positioning.

In this study, participants’ composite scores showed no change across sessions, as illustrated in Figure 3. This finding can likely be attributed to the extended time-gap between sessions and, more importantly, the absence of feedback for participants. Previous research underscores the indispensability of focused feedback in the effective simulation-based training of surgical skills [25]. Further, in this study, the Contrasting Groups method was solely used to define a discriminatory standard to explore supporting evidence for the interpretation of test results. However, we believe that the Contrasting Groups Method should only be used with caution for pass/fail assessments defined as mastery standards [26]. An often-used approach in mastery competence standard setting in simulation-based surgical skills assessment is to define the average score of the experienced group as the pass/fail standard [15]. The simulator test under discussion has been incorporated into a comprehensive iVR simulation for volar plate fixation of a DRF, also including automated performance feedback. In this setting a full score for fluoroscopic control will be obtained only if achieving the average score, or better, than the experienced surgeons in this study. Ongoing studies are exploring the pedagogical merits of this iVR simulator.

### Limitations

First, movement of the operated limb in the simulator was restricted to 3 axes at the elbow and wrist, which prevented movements such as shoulder rotation for obtaining a lateral view where the ulna and radius are aligned and lifting the elbow for a PA view parallel to the anterior inclination of the radiocarpal joint, among others. This was challenging for some of the experienced participants, as it deviated from their usual practice. Second, our research focused solely on intraoperative fluoroscopy control for an already completed surgery, simplifying the real-world application of fluoroscopy, which is predominantly used during the procedure. Third, the iVR simulator’s design inherently eliminates causes of unclear images, such as hand tremor of the surgeon and images not centered over the wrist, as the C-arm in the simulator automatically centers over the operative area, resulting in consistently clear images. These simplifications could facilitate image acquisition, disproportionately aiding the novices. This could contribute to the observed lack of difference between groups in terms of time and number of images taken. Fourth, in the simulated scenario only the operator is present and responsible for obtaining the images, omitting the presence of a radiographer as required in some countries and, hence, the findings cannot necessarily be extended to that setting.

### Conclusion

Our study provided validity evidence from all 5 sources of Messick’s validity framework for an iVR-simulator test to assess performance in intraoperative fluoroscopy control of a DRF fixated by a volar locking plate.

In perspective, our findings suggest that iVR simulation could be a feasible modality for both training and testing of competence in intraoperative fluoroscopy. The simulator test could be used to assess trainees’ fluoroscopic skills in a risk-free environment before proceeding to supervised practice on patients. We propose further investigation of iVR simulation as a potentially valuable tool for trainees to learn the basics of the intricate radiographic anatomy of the distal radius and how to conduct adequate fluoroscopic assessments during surgery. Subsequent future investigations should examine the applicability of skills acquired in the simulated environment to real-world clinical settings and extend the development of similar simulation tools to other joints, anatomical variants, and fixation techniques.

### Supplementary data

A video of a VR session is available as supplementary data on the article page, doi: 10.2340/17453674.2024.41345

## Notes

MS: study design, data collection, data interpretation, writing of the manuscript. AG: study design, data analysis and interpretation, revision of the manuscript. LK: study design, data interpretation, revision of the manuscript. MEJ: study design, data collection, statistical analysis, data analysis and interpretation, revision of the manuscript.

Handling co-editors: Taco Gosens

*Acta* thanks Ruurd Lukas Jaarsma and Charlotte Laane for help with peer review of this manuscript.
